# Supplementary material for: Comparative masticatory myology in anteaters and its implications for interpreting morphological convergence in myrmecophagous placentals
Source: PeerJ. 2020 Sep 3;8:e9690. doi: 10.7717/peerj.9690 (PMC7491420; doi:10.7717/peerj.9690)
Supplement: Table S1 — Abbreviations for the muscle names are as in Table I. NA –not weighted. [file peerj-08-9690-s001.docx]

**Table S1 – Weight, in grams, of the muscles of the six dissected specimens.** Abbreviations for the muscle names are as in Table I. NA – not weighted.

| Muscle | *C. did.*- 1571 | *C. did.* - 1525 | *T. tet.* - 3075 | *T. tet.* - 3074 | *T. tet.* - 778N | *M. tri.* - 3023 |
| --- | --- | --- | --- | --- | --- | --- |
| Mm.m. | 0,0692 | 0,0616 | 0,8789 | 0,9654 | 0,9355 | 6,7043 |
| M.t.s. | 0,0977 | 0,098 | 0,4761 | 0,3158 | 0,4062 | 2,446 |
| pz-M.t.s. | 0,0071 | 0,0085 | 0,0799 | 0,1093 | 0,1237 | 0,7373 |
| pl-M.t.p. | 0,0156 | 0,0466 | 0,1398 | NA | 0,2594 | 0,9061 |
| pm-M.t.p. | 0,0273 | 0,0207 | 0,0794 | 0,0666 | 0,1104 | 0,1783 |
| M.t.p. | 0,0429 | 0,0673 | 0,2192 | NA | 0,3698 | 1,0844 |
| ps-M.p.e. | 0,0084 | 0,0125 | 0,0837 | NA | 0,0745 | 1,2792 |
| pi-M.p.e. | 0,0117 | NA | 0,0319 | NA | 0,0646 | 0,5529 |
| M.p.i. | 0,0389 | NA | 0,3079 | NA | NA | 1,4173 |
| pe-M.b. | 0,0162 | NA | 0,4135 | NA | NA | NA |
| pi-M.b. | 0,0473 | 0,0301 | 1,1378 | NA | 2,3477 | 9,5742 |
| M.ma. | 0,0051 | 0,0127 | 0,0874 | NA | NA | 0,3478 |
| M.i.a. | NA | NA | NA | NA | 0,3211 | NA |
| Mm.mh. | 0,1005 | 0,163 | NA | NA | NA | NA |
