# Supplementary material for: Comparative masticatory myology in anteaters and its implications for interpreting morphological convergence in myrmecophagous placentals
Source: PeerJ. 2020 Sep 3;8:e9690. doi: 10.7717/peerj.9690 (PMC7491420; doi:10.7717/peerj.9690)
Supplement: Table S2 — F-value of F-statistic; R2-adjusted R-squared; DF–degrees of freedom; p–value of p for α = 0.05. [file peerj-08-9690-s002.docx]

**Table S2 – Linear regressions of masticatory and facial-masticatory muscles volume extracted from the stained specimens on the estimated volume based on mass and a density of 1.06 g/cm^-3^.** *F* – value of *F*-statistic; *R^2^* – adjusted *R*-squared; *DF* – degrees of freedom; *p* – value of *p* for α = 0.05.

| **Species** | **ID** | ***F*** | ***R^2^*** | ***DF*** | ***p*** |
| --- | --- | --- | --- | --- | --- |
| *C. didactylus* | M-1571 | 112.70 | 0.93 | 1, 8 | 5.42x10^-6^ |
| *T. tetradactyla* | M-3075 | 64.40 | 0.88 | 1, 8 | 4.27x10^-5^ |
| *M. tridactyla* | M-3023 | 87.46 | 0.93 | 1, 6 | 8.48x10^-5^ |
