# Supplementary figures and images for: Comparative masticatory myology in anteaters and its implications for interpreting morphological convergence in myrmecophagous placentals
Source: PeerJ. 2020 Sep 3;8:e9690. doi: 10.7717/peerj.9690 (PMC7491420; doi:10.7717/peerj.9690)

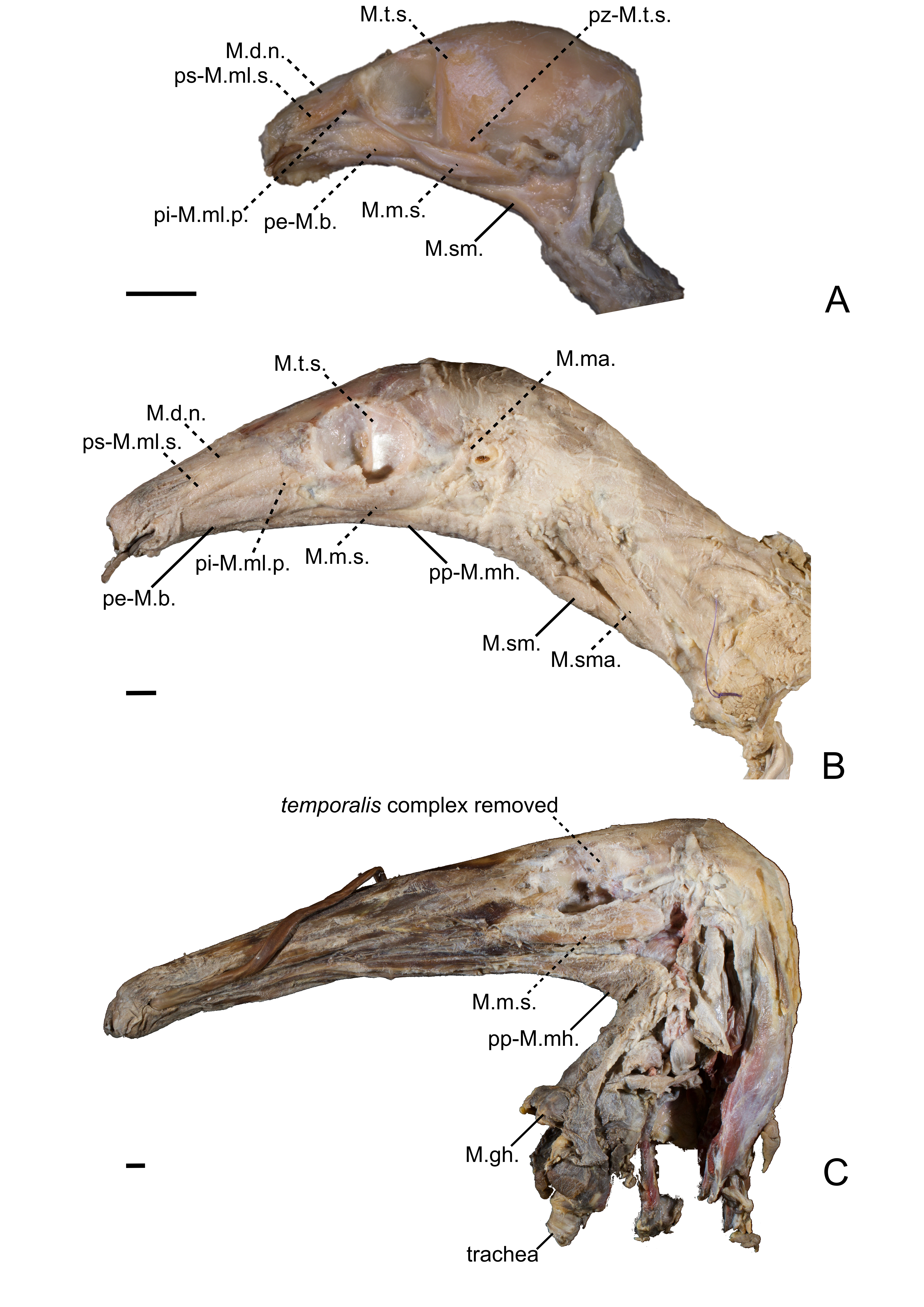

Supplement: Figure S1 — A –Cyclopes didactylus (M1571_JAG); B –Tamandua tetradactyla (M3075_JAG); C –Myrmecophaga tridactyla (M3023_JAG). Scale bar 10 mm. Muscle abbreviations as in Table 1. [file peerj-08-9690-s003.png]

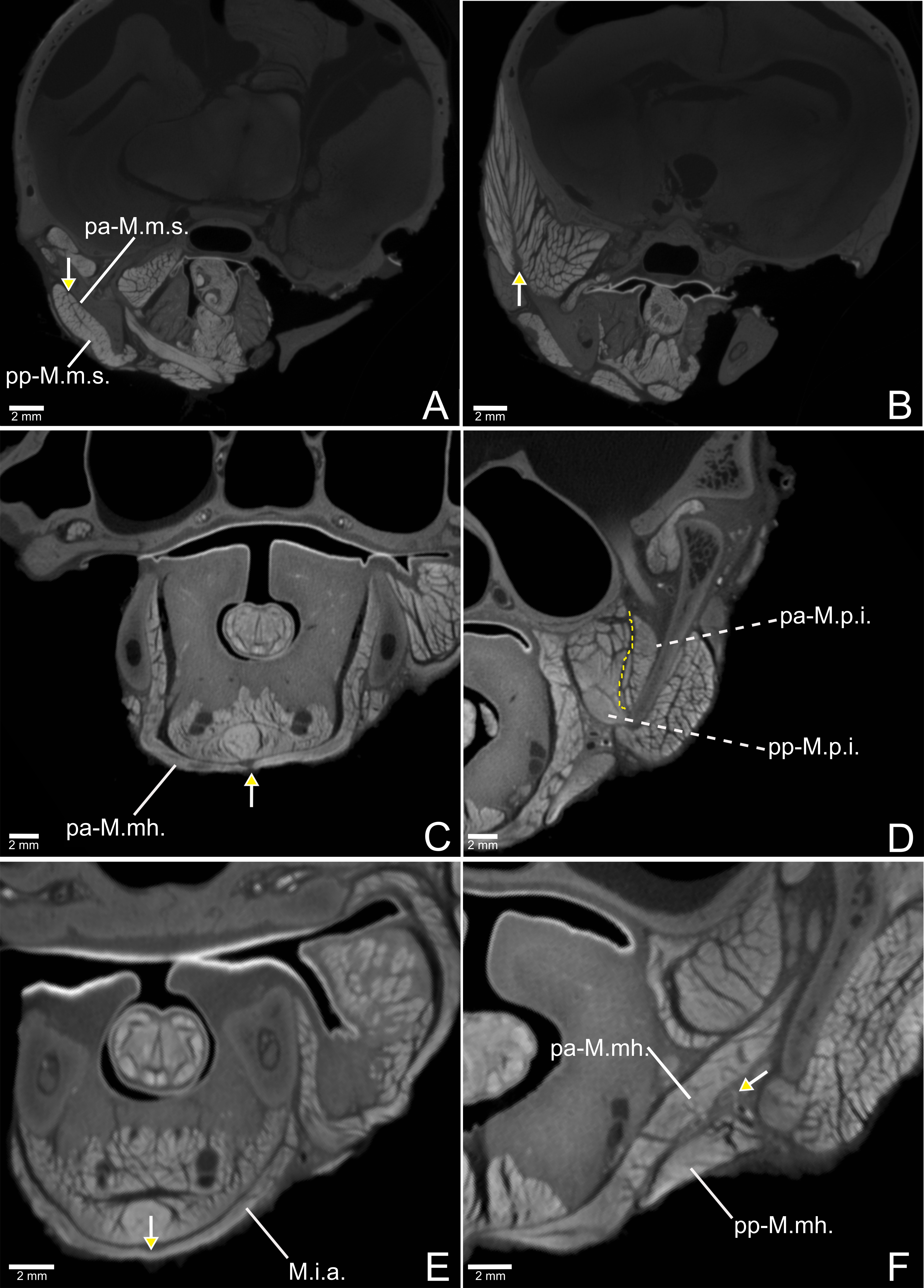

Supplement: Figure S2 — A –the arrow shows the separation between the pars anterior (pa-M.m.s.) and the pars posterior (pp-M.m.s.) of the M. masseter superficialis; B –bipennate architecture of the M. temporalis superficialis and axis of pennation indicated by the arrow; C –the arrow points to the midline raphe of the pars anterior of the M. mylohyoideus (pa-M.mh.); D –the dashed yellow line shows the division between the pars anterior (pa-M.p.i.) and the pars posterior (pp-M.p.i.) of the pterygoideus internus; E –absence of a midline (arrow) raphe on the M. intermandibularis anterior (M.i.a.); F –passage of the sublingual artery splitting the pars anterior (pa-M.mh.) and the pars posterior (pp-M.mh.) of the M. mylohyoideus. [file peerj-08-9690-s004.png]
